# Pro‐inflammatory activation of microglia in the brain of patients with sepsis

**DOI:** 10.1111/nan.12502

**Published:** 2018-06-19

**Authors:** T. Zrzavy, R. Höftberger, T. Berger, H. Rauschka, O. Butovsky, H. Weiner, H. Lassmann

**Affiliations:** ^1^ Center for Brain Research Medical University of Vienna Vienna Austria; ^2^ Clinical Institute of Neurology Medical University of Vienna Vienna Austria; ^3^ Clinical Department of Neurology Medical University of Innsbruck Vienna Austria; ^4^ Department of Neurology Sozialmedizinisches Zentrum Ost‐Donauspital Vienna Austria; ^5^ Karl Landsteiner Institut für neuroimmunologische und neurodegenerative Erkrankungen Donauspital Vienna Vienna Austria; ^6^ Ann Romney Center for Neurological Diseases Brigham and Women's Hospital Harvard Medical School Boston MA USA; ^7^ Evergrande Center for Immunologic Diseases Brigham and Women's Hospital Harvard Medical School Boston MA USA

**Keywords:** brain, microglia activation, perivascular macrophages, sepsis

## Abstract

**Aims:**

Experimental data suggest that systemic immune activation may create a pro‐inflammatory environment with microglia activation in the central nervous system in the absence of overt inflammation, which in turn may be deleterious in conditions of neurodegenerative disease. The extent to which this is relevant for the human brain is unknown. The central aim of this study is to provide an in‐depth characterization of the microglia and macrophage response to systemic inflammation.

**Methods:**

We used recently described markers to characterize the origin and functional states of microglia/macrophages in white and grey matter in patients who died under septic conditions and compared it to those patients without systemic inflammation.

**Results:**

We found pro‐inflammatory microglia activation in septic patients in the white matter, with very little activation in the grey matter. Using a specific marker for resident microglia (TMEM119), we found that parenchyma microglia were activated and that there was additional recruitment of perivascular macrophages. Pro‐inflammatory microglia activation occurred in the presence of homeostatic microglia cells. In contrast to inflammatory or ischaemic diseases of the brain, the anti‐inflammatory microglia markers CD163 or CD206 were not expressed in acute sepsis. Furthermore, we found pronounced upregulation of inducible nitric oxide synthase not only in microglia, but also in astrocytes and endothelial cells.

**Conclusion:**

Our results demonstrate the pronounced effects of systemic inflammation on the human brain and have important implications for the selection of control populations for studies on microglia activation in human brain disease.

## Introduction

Systemic immune activation during the course of peripheral infections affects the central nervous system (CNS), manifesting in signs of the so‐called sickness behaviour including fever, malaise and fatigue 1. This may have deleterious effects creating a pro‐inflammatory state in the CNS, which may exacerbate neurological diseases 2, 3, 4. The underlying pathophysiological mechanisms are still poorly understood. Studies suggest a role for signalling through vagal nerve stimulation and an effect of cytokines on circumventricular organs or the blood–brain barrier which results in increased cytokine production within the brain and spinal cord 1.

Microglial cells, which are the resident brain phagocytes, are believed to play a pivotal role in mediating metabolic and phenotypical changes in the CNS during systemic infection 5, 6. Microglia activation is typically classified into a pro‐inflammatory neurotoxic (M1), and an anti‐inflammatory or neuroprotective (M2) pattern, although this dichotomous division is oversimplified 7, 8. Most data on the communication between the peripheral immune system and the CNS are derived from experimental studies in rodents challenged with pathogens or mimetics of systemic inflammation. A systematic review of 51 studies of animal models reported a direct influence of systemic inflammation on microglial activation, demonstrating activation of microglia cells by upregulation of pro‐inflammatory proteins and concomitant production of pro‐inflammatory cytokines 9. Furthermore, in experimental models of neurodegenerative diseases, systemic inflammation results in disease acceleration and increased neuronal loss, associated with microglia activation and increased production of pro‐inflammatory mediators including IL‐1β, IL‐6 and TNF‐α 10.

Information on microglia phenotypes and activation patterns in humans with peripheral inflammation has not been well studied. To our knowledge, only one study has systematically assessed the microglia phenotype in patients with sepsis in the absence of CNS infection or pathology and used a limited number of markers 11. In our study, we measured leucocyte recruitment and microglia activation in brain tissue devoid of inflammatory, vascular or neurodegenerative pathology in the CNS from patients with systemic sepsis and compared them to carefully selected noninflammatory controls. We found pro‐inflammatory microglia activation primarily in the white compared to grey matter, whereas perivascular recruitment of macrophages was present in both compartments. Molecules with anti‐inflammatory function were only expressed in perivascular macrophages. Interestingly, the expression of the homeostatic microglia marker P2RY12 was similar in septic and control brains. The most prominent difference between patients with sepsis and noninflammatory controls was the massive induction of inducible nitric oxide synthase (iNOS) in microglia, astrocytes and endothelial cells.

## Material and methods

### Sample characterization

**Figure 1:**
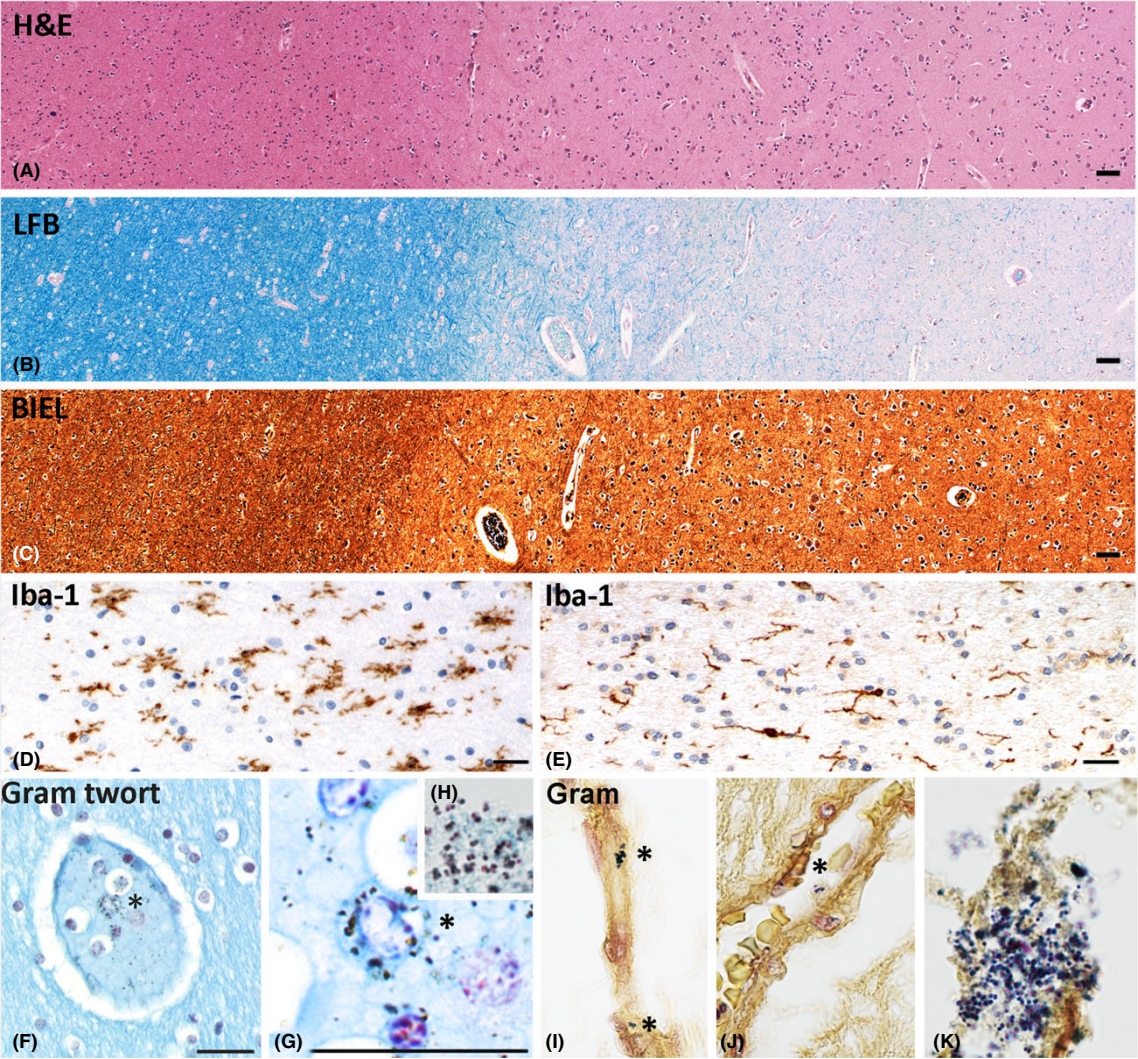
Neuropathological characterization of cases recruited into the study. (**A–C**) Cortex and white matter of a septic patient; no structural brain lesions and no signs of inflammation are seen in sections stained with haematoxylin and Eosin (**H**&**E**;** A**), luxol fast blue myelin stain (LFB;** B**) and Bielschowsky silver impregnation (BIEL;** C**). (**D**,**E**) Microglia phenotype in sections stained with the pan‐microglia marker Iba‐1; in the white matter of a septic patient (**D**) microglia are enlarged and show an amoeboid phenotype in contrast to the ‘resting’ phenotype seen in the white matter of a control patient (**E**). (**F–K**) In the white matter of a patient with sepsis, macrophages containing gram positive bacteria are seen in the vessel lumen (*), but there is no evidence for gram positive bacteria in the perivascular spaces or the parenchyma. As a positive control we included the staining of bacteria within a bacterial abscess (**H** and **K**). Magnification bars: 50 μm.

Our study was performed on archival paraffin embedded autopsy tissue collected in the Center for Brain Research and at the Institute of Neurology at the Medical University of Vienna. It included 10 patients, who died under septic conditions and 11 control patients, who died without clinical evidence of systemic infection or sepsis (Table 1). All available medical records, laboratory charts and autopsy charts were screened. Septic patients had to fulfil the following criteria: clinical diagnosis of sepsis by the physician (with microbiological confirmation available in 8/10 patients) 12. None of the patients suffered from neurological disease and a detailed neuropathological evaluation did not provide evidence for an inflammatory or neurodegenerative disease (Figure 1 **A–C**). All tissue sections from the septic patients were screened with Gram and Gram Twort staining for bacteria and no perivascular bacteria were seen. However, all sections from septic patients contained at least some intravascular macrophages with bacteria (Figure 1 **F–K**). This was also seen in the two patients, where microbiological confirmation of bacterial sepsis was not available. Control patients had a cause of death unrelated to systemic inflammation or sepsis. Two patients in the control cohort suffered from Parkinson's disease, however, the tissue blocks of temporal cortex and white matter included in our study were devoid of any α−synuclein positive Lewy‐bodies. Furthermore, detailed analysis of sections with haematoxylin & eosin, luxol fast blue and Bielschowsky silver impregnation did not reveal pathological alterations in the grey and white matter.

**Table 1:** Clinical demographics and microbiology of cases included in the study

Case	Details	Sex	Age	Cause of death	Microbiology	Intravascular bacteria
Control 1	Control	female	27	Suicide		
Control 2	Control	female	35	pulmonary embolism		
Control 3	Control	female	39	cervix CA		
Control 4	Control	female	42	lung CA		
Control 5	Control	male	46	pulmonary embolism		
Control 6	Control	male	65	myocardial infarction		
Control 7	Control	female	71	myocardial infarction		
Control 8	Control	male	72	cardiovascular failure		
Control 9	Control	male	83	cardiovascular failure		
Control 10	Control	female	88	cardiovascular failure		
Control 11	Control	male	71	myocardial infarction		
Septic 1	Septic	female	21	multiple organ failure	Staphylococcus	+
Septic 2	Septic	male	23	septic shock	Staphylococcus	++
Septic 3	Septic	female	38	multiple organ failure	Escherichia	++
Septic 4	Septic	male	40	cardiac arrest	Staphylococcus	++
Septic 5	Septic	female	51	cardiovascular failure	n.a.	++
Septic 6	Septic	male	56	organ failure	Staphylococcus	++
Septic 7	Septic	female	58	septic shock	Neisseria	++
Septic 8	Septic	male	66	septic shock	Staphylococcus	+
Septic 9	Septic	male	83	myocardial infarction	Proteus; Klebsiella; Enterococcus	++
Septic 10	Septic	female	89	cardiovascular failure	n.a.	+

CA, carcinoma; n.a., not available.

Intravascular bacteria: semiquantitative rating of macrophages with gram positive bacteria in their cytoplasm; +: single vessels with macrophages; ++: some vessels with macrophages containing bacteria.

### Microglia and Macrophage Markers

Immunohistochemistry was performed on paraffin sections with a biotin/avidin detection system as described in detail before 13, 14 using markers defined in Table 2. For the characterization of microglia and macrophages, the general marker Iba‐1 was used to depict all microglia and macrophages. To distinguish between macrophages recruited from the periphery and resident microglia we employed TMEM119. TMEM119 is a transmembrane protein expressed on microglia but not on recruited blood derived macrophages 15, 16. For resting (homeostatic) microglia we used P2RY12, a microglia antigen, which is rapidly downregulated in microglia in experimental rodent models and human brain tissue in inflammatory and neurodegenerative diseases 17, 18. CD68 was used as a marker involved in phagocytosis, MHC Class I and Class II and the costimulatory molecule CD86 as markers for antigen presentation, and p22phox as part of the NADPH oxidase complex as well as iNOS to depict oxygen and nitric oxide radical production in macrophages or microglia. As prototypical markers associated with anti‐inflammatory function of macrophage populations, we used the haptoglobin/haemoglobin scavenger receptor CD163 and the mannose receptor CD206 19. As CD206 and CD163 are constitutively expressed on perivascular macrophages in the normal brain we made use of these markers to compare the quantity of perivascular macrophages in this study. All markers have been extensively characterized regarding their reliability and expression patterns in human autopsy material of lymphatic tissue 18. For evaluation of lymphocytic infiltration, CD3 was used to assess all T cells. Transglutaminase 2 (TG2) and glial fibrillary acidic protein (GFAP) was used as an endothelial and astrocyte marker for double staining.

**Table 2:** Primary antibodies and immunocytochemical techniques

#	Antibody	Origin	Target	Dilution	Antigen retrieval	Source
1	CD3	Rabbit (mAB)	T‐cells	1:2000	St (E)	RM‐9107‐S; Neomarkers
2	Iba‐1	Rabbit (pAB)	Ionized calcium binding adaptor molecule 1	1:3000	St (E)	019‐19741; Wako
3	CD68	Mouse (mAB; IgG1)	CD68 110‐kD transmembrane glycoprotein in macrophages	1:100	St (E)	M0814; Dako,
4	HLA‐DR	Mouse (mAB; IgG1)	MHC Class II antigen	1:100	St (C)	M0775; Dako,
5	p22phox	Rabbit (pAB)	NADPH oxidase protein	1:100	St (C)	sc‐20781; Santa Cruz
6	TMEM119	Rabbit (pAB)	Transmembrane protein 119	1:100	‐	HPA051870; Sigma‐Aldrich
7	P2RY12	Rabbit (pAB)	Purinergic receptor	1:2500	St (E)	Harvard, Dr. Butovsky
8	HC10	Mouse (mAB; IgG2a)	Heavy chain of MHC Class I	1:2000	St (E)	Stam *et al*., 1990
9	Ferritin	Rabbit (pAB)	Iron storage protein	1:1000	St (E)	MO (F5012); Sigma‐Aldrich
10	CD206	Mouse (mAB; IgG1)	Mannose receptor	1:100	St (E)	ab117644; abcam
11	CD163	Mouse (mAB; IgG1)	Haemoglobin‐haptoglobin scavenger receptor	1:1000	St (C)	NCL‐CD163; Novocastra
12	iNOS	Rabbit (pAB)	Inducible nitric oxide synthase I	1:200	St (E)	PA1‐37925; Thermo Scientific
13	CD86	Goat (pAB)	Costimulatory T‐cell signal	1:250	St (C)	AF‐141‐NA; R&D Systems
14	GFAP	Rabbit (pAB)	Glial fibrillary acidic protein	1:3000	St(E)	Z0334; Dako
15	TG2	Mouse (mAB)	Transglutaminase 2	1:5000	St (E)	AB‐3; NeoMarkers

MAB, monoclonal antibody; pAB, polyclonal antibody; ST (E), antigen retrieval by steaming in EDTA buffer (pH: 9); St(C), antigen retrieval by steaming in citrate buffer (pH: 5).

### Immunohistochemistry

Sections were routinely deparaffinized followed by blocking of the endogenous peroxidase with methanol and 0.02% H_2_O_2_. Antigen retrieval was done as outlined in Table 2. Nonspecific protein binding was blocked by incubation with a mixture of 10% foetal calf serum and DAKO buffer. Primary antibodies were applied overnight at 4°C at the dilutions indicated in Table 2. Antibody labelling was detected by using species‐specific biotinylated secondary antibodies against mouse, rabbit or goat immunoglobulins, incubation with streptavidin/peroxidase complex and development with diaminobenzidine. For control, immunohistochemistry was performed in the absence of the primary antibodies and by using normal rat and goat serum or isotype‐matched monoclonal antibodies.

For double staining using primary antibodies derived from different species, the same antigen retrieval techniques and incubation with primary antibodies was used as described above. Antibody binding was visualized with either biotinylated secondary antibodies and peroxidase‐ or alkaline phosphatase‐conjugated streptavidin or directly with horseradish peroxidase (HRP) or alkaline phosphatase conjugated secondary antibodies (MACH 4™; Biocare Medical). Reaction products were visualized by development with fast blue BB salt (for alkaline phosphatase; blue) or amino ethyl carbazole (AEC; for peroxidase; red) respectively.

Double staining using antibodies derived from same species (Iba‐1, P2RY12, iNOS, TMEM119, p22phox, GFAP) were performed with a different protocol by using heat‐induced epitope retrieval between the subsequent immunohistochemical reactions 14. After deparaffinization and a primary round of antigen retrieval sections were incubated with the primary antibody (TMEM119/P2RY12/iNOS), followed by biotinylated anti‐rabbit antibody. In the case of P2RY12 and iNOS catalysed signal amplification with avidin peroxidase followed by biotinylated tyramine was performed. Finally, streptavidin alkaline phosphatase was applied and the reaction was visualized by development with fast blue BB salt (blue). Then, another round of antigen retrieval for 30 min at pH 9 was performed, which abolishes antibody reactivity from the previous round but leaves the visualized product intact. Afterwards, the second immunohistochemistry reaction was applied using Iba‐1/p22phox/GFAP/CD68 antibody as the primary antibody and the biotinfree horseradish peroxidase (HRP) antibody conjugated system (MACH 4™; Biocare Medical) as detection system. Peroxidase reaction products were visualized using amino ethyl carbazole (AEC; red). For control reasons, immunohistochemistry was performed in the absence of the second primary antibodies confirming inactivation of the first peroxidase step by heat‐induced epitope retrieval. In addition, double staining of P2RY12 and GFAP confirmed absence of colocalization.

#### Immunofluorescence

Since both primary antibodies come from the same species (rabbit), double staining for iNOS and Iba‐1 was performed using extensive heat‐induced epitope retrieval between the subsequent immunohistochemical reactions. After deparaffination and a primary round of antigen retrieval, sections were incubated with iNOS followed by biotinylated anti‐rabbit antibody, avidin peroxidase and catalysed signal amplification with biotinylated tyramine. Then, another round of antigen retrieval for 30 min at pH 9 was performed. Afterwards DyLight 549 Anti‐Streptavidin was applied. Later, the second immunohistochemistry reaction was applied using Iba‐1 antibody as the primary antibody and anti‐rabbit Cy2 as a secondary antibody. Immunofluorescence was visualized in a confocal laser microscope (Leica SP2).

### Quantitative evaluation

We manually quantified white and grey matter areas of temporal lobe tissue. The number of T cells were counted separately in the perivascular and parenchymal areas. Later, these values were pooled in the statistical analysis. For quantification of lymphocytes, a morphometric grid within the ocular lens was used and 30 fields of 0.9126 mm^2^ per tissue were counted.

For the quantitative evaluation of microglia/macrophages, sections were overlaid by a morphometric grid (0.2256 mm^2^) placed within the ocular lens and parenchymal cells were quantified in 2 to 3 fields of the white and grey matter. Cells expressing the respective marker were counted and the values were expressed as cell counts per square millimetre. INOS‐positive microglia were identified by their morphological appearance and the microglia expression was confirmed by double staining with Iba‐1. We counted CD206^+^ and CD163^+^ perivascular cells to quantify perivascular macrophages by counting 10 fields in the white and grey matter separately.

Digital optical densitometry was performed for markers, which were not exclusively expressed in macrophages and microglia, such as the MHC Class I marker HC10, ferritin and iNOS according to a previously published protocol 20. Expression of these markers was quantified by calculating the positive DAB signal area fraction using ImageJ. One to two images per region of interest were taken at 10× objective lens magnification (0.43 mm^2^). Images were saved as TIFF. For digitally removing haematoxylin counterstaining, a colour deconvolution plugin (freeware kindly provided from A. C. Ruifrok, NIH) was run. Furthermore, RGB images were converted into 8‐bit grey scale images and inverted. A threshold was set in resulting images and the area fraction was calculated. All values are expressed as percentage of positive area.

### Statistical analysis

Statistical analysis was performed with IBM SPSS 21 and GraphPad Prism 6. Due to uneven distribution of our data, statistical analysis was performed with nonparametric tests. Descriptive analysis included median value and range. Differences between two groups were assessed with Wilcoxon–Mann–Whitney U‐test. Correction for multiple testing was performed according to Bonferroni–Holm procedure. Interdependence of variables was evaluated by the Spearman nonparametric correlation test. The reported *p*‐values are results of two‐sided tests. A *P*‐value ≤ 0.05 was considered statistically significant.

## Results

**Figure 2:**
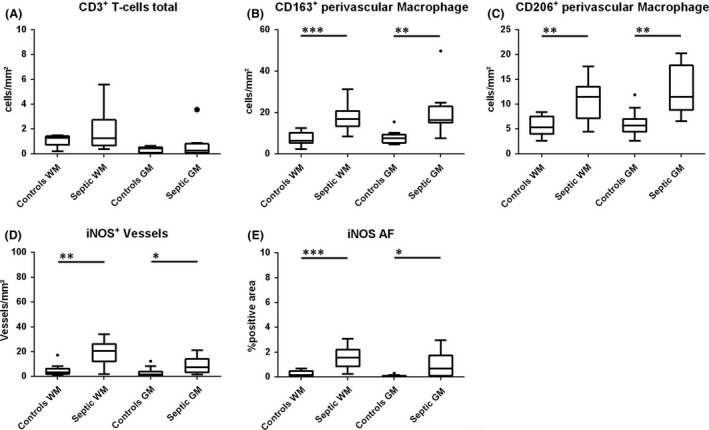
Quantitative evaluation of CD3^+^ T‐cells (**A**), CD163^+^ or CD206^+^ perivascular macrophages (**B,C**) and iNOS expression in vessels (**D**) or in the global tissue determined by densitometry (**E**). WM: white matter; GM: grey matter.

The aim of our study was to investigate the effect of systemic infection on the activation patterns of microglia and recruited macrophages in the human CNS. We selected autopsy material from patients with sepsis, confirmed by clinical microbiology, and compared them to patients, in whom a systemic inflammatory condition at the time of death could be excluded (Table 1). Since in two septic patients, microbiological confirmation of sepsis was not available, we additionally analysed the sections for bacteria containing intravascular macrophages using Gram staining and found them in variable numbers in all septic patients (Table 1; Figure 1 **F–K**). The mean age of the inflammatory cohort was 52.5 years (range 21 to 89), which was slightly lower than in the control cohort (58 years, range 27 to 88). Gender distribution was nearly equal between septic and nonseptic patients (female 6:5; male 5:5). Since microglia are activated in the course of brain inflammation or neurodegeneration, we selected tissue blocks which were carefully screened for the absence of pathological changes, visible in sections stained with haematoxylin & eosin, luxol fast blue myelin staining and Bielschowsky silver impregnation (Figure 1 **A–C**). Specifically, no inflammatory infiltrates were seen in the sections in haematoxylin & eosin stained sections. As described previously 21 very few perivascular or parenchymal CD3^+^ T‐cells were present and their number in the perivascular space and the parenchyma was not significantly different in septic vs. noninflammatory patients, both in the cortex and white matter (Figure 2 **A**). Granulocyte infiltration was absent in both control and septic patients. The only global difference between septic and control tissue was a more pronounced microglia activation phenotype, the cells being enlarged with partly increased and partly retracted processes in the former (Figure 1 **D**,**E**).

### Microglia and macrophage phenotype in nonseptic controls:

**Figure 3:**
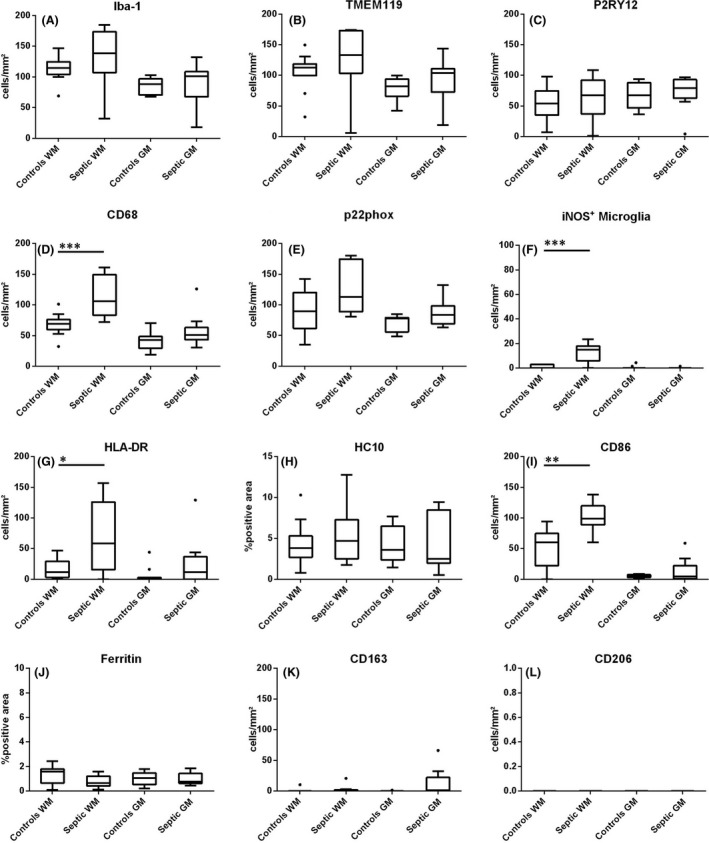
Quantitative profiles of microglia activation in the cortex and white matter of septic patients in comparison to noninflammatory controls for the microglia and macrophage marker Iba‐1 (**A**), the specific microglia marker TMEM119, (**B**) the ‘homeostatic’ microglia marker P2RY12 (**C**) and the microglia activation markers CD68 (**D**), p22phox (**E**), iNOS (**F**), HLA‐D (**G**), MHC Class I (HC10; H), CD86 (**I**), ferritin (**J**), CD163 (**K**) and CD206 (**L**). The values represent cells/mm^2^ or the area fraction (AF) determined by densitometry.

The white matter (WM) contained significantly more Iba‐1^+^ and TMEM119^+^ microglia compared to cortex (GM) (114.4 *vs*. 88; 112.9 *vs. 82.1; P* = 0.000; *P* = 0.005 respectively) (Figure 3 **A**,**B**). More than 90% of all microglia‐like cells coexpressed Iba‐1 and the microglia‐specific marker TMEM119 (98% of Iba‐1 + cells in the WM and 93% in the GM). In addition, significantly more microglia in the white matter expressed the phagocytosis marker CD68 (*P* = 0.001) and the T‐cell costimulatory molecule CD86 (*P* = 0.005) as compared to the cortex (Figure 3 **D**,**I**). In comparison to data published in rodents 17, 22, microglia in the human CNS showed a partially preactivated phenotype as seen by a reduced number of P2RY12^+^ microglia (47.43% of Iba‐1 + cells in the WM and 76.6% in the GM) and a profound expression of p22phox and CD68 (78% and 60% (WM); 88% and 48% (GM) (Figure 3 **C**,**E**). iNOS expression was largely absent in microglia (Figure 3 **F**), however, it was found in some immunoreactive endothelial cells. Neither significant age‐related correlations nor gender‐specific differences in microglia activation patterns were seen. CD163 and CD206 were only expressed in perivascular macrophages (Figure 3 **K**,**L)**.

### Microglia in the white matter of septic patients:

**Figure 4:**
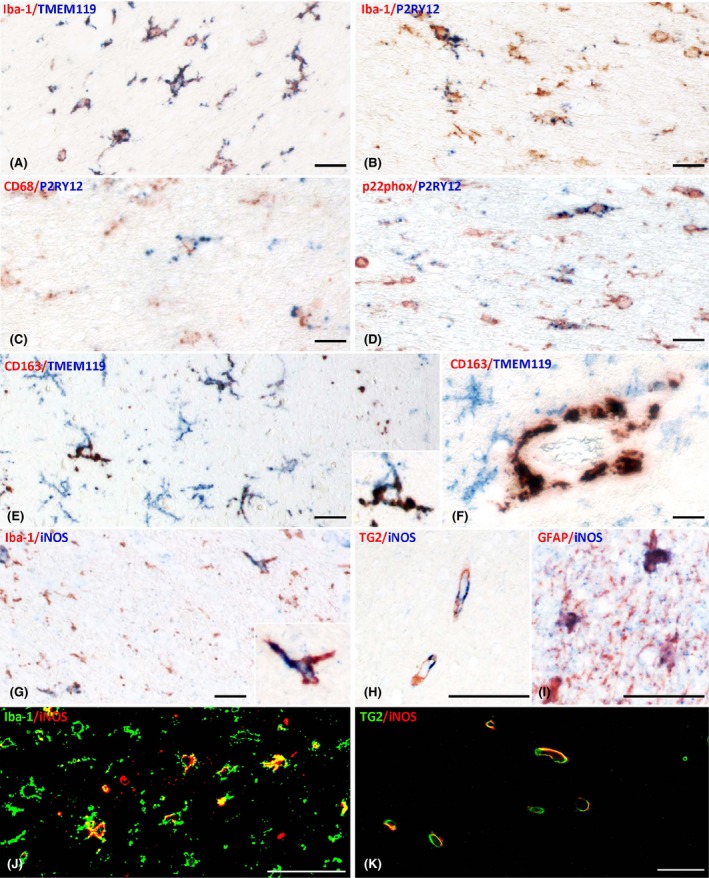
Patterns of microglia activation shown by double staining. (**A–D**) Coexpression of different markers in the white matter of a patients with sepsis shows that nearly all Iba‐1 positive cells also express TMEM119 **(A)**, but only a fraction is P2RY12 positive (**B**); despite the expression of the ‘homeostatic’ marker P2RY12 some microglia also express the activation markers CD68 (**C**) of p22phox (**D**). (**E–F**) When CD163 is present in cells with microglia morphology they are also stained for TMEM119 (**E** and insert); in contrast, perivascular macrophages are CD163^+^ but TMEM119 negative (**F**). (**G–K**) iNOS is expressed in microglia (**G,J**) and astrocytes (**I**) in addition in endothelial cells in sepsis as well as in controls (**H,K**). Magnification Bars: 50 μm.

Similar to control patients, nearly all cells with a morphological microglia phenotype (96%) coexpressed Iba‐1 and TMEM119 (Figure 4 **A**). In general, microglia cells were more likely to be enlarged, with increased or retracted cell processes and some showed an amoeboid phenotype (Figure 1 **D**,**E)**. There was no additional loss of the homeostatic marker P2RY12 compared to controls (Figures 3 **C** and 4 **B**) and microglia coexpressed P2RY12 and pro‐inflammatory markers (Figure 4 **C**,**D**). However, the expression profile of pro‐inflammatory molecules was significantly different. Compared to controls, we found a strong increase of the phagocytosis marker CD68 (*P* = 0.000) and of both CD86 and HLA‐DR (*P* = 0.002 and *P* = 0.049, respectively) (Figure 3 **D**,**G**,**I**). Likewise, iNOS expression on cells with a microglia morphology was significantly higher compared to controls (*P* = 0.004) and a trend towards higher numbers of p22phox positive cells was seen (Figure 3 **E**,**F**). In addition, the number of iNOS‐positive vessels was significantly elevated (*P* = 0.004) and reactivity was also present in astrocytes (Figure 2 **D**; Figure 4 **G–K**). To evaluate the overall expression of iNOS, we determined the immunoreactive area fraction by densitometry, which was significantly higher (*P* = 0.000) as well (Figure 2 **E**).

The expression of the putative anti‐inflammatory molecules CD163 and CD206 on parenchymal microglia was sparse or absent and not different between septic patients and nonseptic controls (Figure 3 **K**,**L**). However, the numbers of CD163^+^ and CD206^+^ perivascular macrophages were significantly increased compared to the control cohort (*P* = 0.000 [CD163]; *P* = 0.003 [CD206]; Figure 2 **B**,**C**).

### Microglia in the grey matter of septic patients:

Overall, changes in microglia activation patterns in the cortex of septic patients in comparison to controls were subtle and immunoreactivities more resembled the control cohort than the white matter of septic patients. All microglia coexpressed Iba‐1^+^ and TMEM119^+^. We did not observe a difference in the expression of the homeostatic marker P2RY12, which was found on 81% of the Iba‐1^+^ microglia cells in the grey matter of septic patients (Figure 3 **A–C**).

The expression profile of molecules associated with pro‐inflammatory activity did not reach statistical significance although trends for a higher expression level of CD68 and p22phox were observed (*P* = 0.095; *P* = 0.092, Figure **D**,**E**). iNOS expression on microglia was similar to controls (Figure 3 **F**), however, significantly more vessels showed immunoreactivity for iNOS (*P* = 0.02) (Figure 2 **D**). None of the microglia cells in the cortex expressed CD206 and this was also the case for CD163 with the exception of two cases, where a moderate number of CD163^+^/TMEM119^+^ double positive microglia cells were present (Figure 4 **E**). Similarly, as observed in the white matter, the number of perivascular macrophages was increased in septic patients in comparison to controls and did not coexpress TMEM119^+^ (CD163: *P* = 0.001; CD206: *P* = 0.002 Figure 2 **B**,**C**; Figure 4 **F**).

No significant differences were observed in the expression of the microglia activation marker ferritin between white and grey matter or between septic or control patients (Figure 3 **J**).

## Discussion

Our study shows that systemic immune activation in humans during sepsis results in pro‐inflammatory activation of resident microglia and a moderate increase in perivascular macrophages in the absence of significant recruitment of lymphocytes or granulocytes into the human brain tissue. These findings are consistent with those reported in experimental animals, which showed that peripheral bacterial or lipopolysaccharide (LPS) challenges, mimicking peripheral inflammation, lead to microglia activation. In models of neurodegenerative diseases, microglia play an important role in neuronal loss and disease progression following systemic inflammation 9, 23. This process appears to have a negative impact on CNS function and can lead to aggravation of neurological diseases 24, 25. However, our data also reveal differences in the patterns of microglia activation between humans and rodents. While in the normal brain and spinal cord of rodents, all microglia show a homeostatic phenotype, and do not express any activation markers, this is not the case in the normal grey and white matter of humans 17, 18, 21. Our study identifies systemic inflammation as one potential factor driving microglia activation in human brain. However, microglia activation was also seen in the brain of patients without systemic inflammation. Reasons for this preactivation may be related to brain ageing and age‐related neurodegeneration, terminal hypoxic conditions, toxins and lifestyle 10, 26. Recently, gene expression analysis showed that rodent and human microglia have a similar core expression pattern, however, during ageing, more genes are regulated in an opposing rather than in an overlapping fashion 27. We could not detect significant correlations with age, which might be attributable to the small sample size.

Intravenous LPS administration to healthy humans leads to microglia activation as demonstrated by the application of the PET ligand TSPO 28. However, TSPO is not specific for a defined functional state of microglia and is also expressed in astrocytes 29. By determining the expression of functionally defined molecules, we provide evidence that increased microglia activation in the white matter is characterized by an upregulated protein expression of the phagocytosis‐associated molecule CD68. Similar findings related to CD68 expression have been reported in a previous study 11. In addition, systemic inflammation may increase the ability of microglia to interact with T cells due to concomitant upregulation of major histocompatibility complex (MHC) and costimulatory molecules. The high expression of NADPH oxidase‐related molecules and iNOS suggests that these activated microglia may be cytotoxic through the production of reactive oxygen and nitric oxide species. The production of reactive oxygen and nitrogen species during sepsis is proposed as a molecular mechanism for septic encephalopathy, cognitive disturbances and neurodegeneration 30. Furthermore, iNOS expression was not restricted to microglia cells but was significantly increased in astrocytes and endothelial cells. Endothelial iNOS expression was previously associated with neuronal damage in the brain in patients dying from septic shock. Prolonged activation of endothelial cells might lead to microcirculatory dysfunction and subsequently to a predisposition for ischaemic lesions, which are known to occur in septic patients 31, 32, 33.

In contrast, markers for anti‐inflammatory activity such as CD163 and CD206, were detected on microglia at low levels and were not differently expressed between septic and control patients. This is different from the microglia activation patterns in multiple sclerosis 18, 34 or stroke lesions 21, where in established lesions the majority of microglia show an intermediate phenotype with the simultaneous expression of pro‐ and anti‐inflammatory markers. One possible explanation for this observation is that in our present study all patients died during the acute phase of sepsis. It is thus far unresolved whether microglia convert to a homeostatic phenotype or remain in an intermediate state of activation, similar to that seen in chronic inflammatory diseases, when patients have survived the acute phase of sepsis. However, both molecules are constitutively expressed on perivascular macrophages and we found an increase of macrophages in the perivascular space. The absence of TMEM119 expression on these cells suggests that they are derived from recruited myeloid cells and not from the resident microglia pool. Perivascular macrophages in peripheral inflammatory conditions are implicated in driving the acute stress responses via synthesis of prostaglandins 35.

Studies suggest that the loss of homeostatic markers in microglia is the first step in their activation, which is triggered by inflammation as well as by neurodegeneration 17, 36, 37. The purinergic receptor P2RY12, sensing ADP release from damaged tissue and being necessary for rapid migration of cells to the site of tissue damage 38, is one of the key markers for the homeostatic phenotype of microglia. In line with this is the nearly complete loss of P2RY12 expression on microglia in active experimental and human inflammatory and neurodegenerative lesions 36, 37. However, it has been shown that P2RY12 is lost in a major subpopulation of microglia in the normal human brain and it was a surprise that systemic immune activation in sepsis did not lead to a further downregulation of its expression in microglia. The presence of TMEM119 reactivity in P2RY12 negative microglia suggests that loss of this purinergic receptor is not due to the recruitment of circulating myeloid cells, which differentiate into a microglia‐like morphological phenotype. We have shown previously that the loss of P2RY12 reactivity correlates with the extent of neurodegeneration in human cortex of controls and Alzheimer's disease patients 36. Thus, neurodegeneration instead of inflammation may be the key trigger for the downregulation of P2RY12 expression on CNS microglia.

In summary, our study provides a comprehensive characterization of the microglia activation pattern associated with systemic immune activation during sepsis. Since systemic inflammation is a frequent complication in patients with terminal disease, these findings must be considered when investigating potential immune mediated mechanisms of tissue damage in human brain disease. Our study further supports the concept that systemic immune activation may play a role in amplifying tissue injury in neurodegenerative diseases 10, but that the extent of these effects may be different in the grey and white matter of the CNS.

## Author contributions

H.L.: design of the study and supervision of all immunocytochemical and quantitative data generation; T.Z. generation of all quantitative data; R.H. neuropathological case selection; T.B. and H.R. generation of clinical data; O.B. and H.W. expertise on microglia biology, design of the study; critical evaluation and discussion of the results. All authors have extensively contributed to the preparation of the manuscript and the interpretation of the findings.

## References

[1] Dantzer R . Cytokine‐induced sickness behaviour: a neuroimmune response to activation of innate immunity. Eur J Pharmacol 2004; 500: 399–411 15464048 10.1016/j.ejphar.2004.07.040

[2] Umemura A , Oeda T , Tomita S , Hayashi R , Kohsaka M , Park K , Sugiyama H , Sawada H . Delirium and high fever are associated with subacute motor deterioration in Parkinson disease: a nested case‐control study. PLoS ONE 2014; 9: e94944 24887491 10.1371/journal.pone.0094944 PMC4041721

[3] Holmes C , Cunningham C , Zotova E , Woolford J , Dean C , Kerr S , Culliford D , Perry VH . Systemic inflammation and disease progression in Alzheimer disease. Neurology 2009; 73: 768–74 19738171 10.1212/WNL.0b013e3181b6bb95 PMC2848584

[4] Buljevac D , Flack HZ , Hop WCJ , Hijdra D , Laman JD , Savelkoul HFJ , van der Meche FGA , van Doorn PA , Hintzen RQ . Prospective study on the relationship between infections and multiple sclerosis exacerbations. Brain 2002; 125: 952–60 11960885 10.1093/brain/awf098

[5] Riazi K , Galic MA , Kuzmiski JB , Ho W , Sharkey KA , Pittman QJ . Microglial activation and TNFalpha production mediate altered CNS excitability following peripheral inflammation. Proc Natl Acad Sci U S A 2008; 105: 17151–6 18955701 10.1073/pnas.0806682105 PMC2579393

[6] Perry VH , Teeling J . Microglia and macrophages of the central nervous system: the contribution of microglia priming and systemic inflammation to chronic neurodegeneration. In Seminars in immunopathology: Springer, 2013; 601–12 10.1007/s00281-013-0382-8 PMC3742955 23732506

[7] Ransohoff RM . A polarizing question: do M1 and M2 microglia exist? Nat Neurosci 2016; 19: 987–91 27459405 10.1038/nn.4338

[8] Walker DG , Lue LF . Immune phenotypes of microglia in human neurodegenerative disease: challenges to detecting microglial polarization in human brains. Alzheimers Res Ther 2015; 7: 56 26286145 10.1186/s13195-015-0139-9 PMC4543480

[9] Hoogland IC , Houbolt C , van Westerloo DJ , van Gool WA , van de Beek D . Systemic inflammation and microglial activation: systematic review of animal experiments. J Neuroinflammation 2015; 12: 114 26048578 10.1186/s12974-015-0332-6 PMC4470063

[10] Perry VH , Holmes C . Microglial priming in neurodegenerative disease. Nat Rev Neurol 2014; 10: 217–24 24638131 10.1038/nrneurol.2014.38

[11] Lemstra AW , Groen in't Woud JC , Hoozemans JJ , van Haastert ES , Rozemuller AJ , Eikelenboom P , van Gool WA . Microglia activation in sepsis: a case‐control study. J Neuroinflammation 2007; 4: 4 17224051 10.1186/1742-2094-4-4 PMC1783646

[12] Levy MM , Fink MP , Marshall JC , Abraham E , Angus D , Cook D , Cohen J , Opal SM , Vincent JL , Ramsay G , Sccm/Esicm/Accp/Ats/Sis . 2001 SCCM/ESICM/ACCP/ATS/SIS International Sepsis Definitions Conference. Crit Care Med 2003; 31: 1250–6 12682500 10.1097/01.CCM.0000050454.01978.3B

[13] Fischer MT , Wimmer I , Hoftberger R , Gerlach S , Haider L , Zrzavy T , Hametner S , Mahad D , Binder CJ , Krumbholz M , Bauer J , Bradl M , Lassmann H . Disease‐specific molecular events in cortical multiple sclerosis lesions. Brain 2013; 136: 1799–815 23687122 10.1093/brain/awt110 PMC3673462

[14] Bauer J , Lassmann H . Neuropathological techniques to investigate central nervous system sections in multiple sclerosis. Methods Mol Biol (Clifton, NJ) 2016; 1304: 211–29 10.1007/7651_2014_151 25520281

[15] Satoh J , Kino Y , Asahina N , Takitani M , Miyoshi J , Ishida T , Saito Y . TMEM119 marks a subset of microglia in the human brain. Neuropathology 2016; 36: 39–49 26250788 10.1111/neup.12235

[16] Bennett ML , Bennett FC , Liddelow SA , Ajami B , Zamanian JL , Fernhoff NB , Mulinyawe SB , Bohlen CJ , Adil A , Tucker A , Weissman IL , Chang EF , Li G , Grant GA , Hayden Gephart MG , Barres BA . New tools for studying microglia in the mouse and human CNS. Proc Natl Acad Sci U S A 2016; 113: E1738–46 26884166 10.1073/pnas.1525528113 PMC4812770

[17] Butovsky O , Jedrychowski MP , Moore CS , Cialic R , Lanser AJ , Gabriely G , Koeglsperger T , Dake B , Wu PM , Doykan CE , Fanek Z , Liu L , Chen Z , Rothstein JD , Ransohoff RM , Gygi SP , Antel JP , Weiner HL . Identification of a unique TGF‐beta‐dependent molecular and functional signature in microglia. Nat Neurosci 2014; 17: 131–43 24316888 10.1038/nn.3599 PMC4066672

[18] Zrzavy T , Hametner S , Wimmer I , Butovsky O , Weiner HL , Lassmann H . Loss of ‘homeostatic’ microglia and patterns of their activation in active multiple sclerosis. Brain 2017; 140: 1900–13 28541408 10.1093/brain/awx113 PMC6057548

[19] Martinez FO , Gordon S . The M1 and M2 paradigm of macrophage activation: time for reassessment. F1000Prime Rep 2014; 6: 13 24669294 10.12703/P6-13 PMC3944738

[20] Hametner S , Wimmer I , Haider L , Pfeifenbring S , Bruck W , Lassmann H . Iron and neurodegeneration in the multiple sclerosis brain. Ann Neurol 2013; 74: 848–61 23868451 10.1002/ana.23974 PMC4223935

[21] Zrzavy T , Machado‐Santos J , Christine S , Baumgartner C , Weiner HL , Butovsky O , Lassmann H . Dominant role of microglial and macrophage innate immune responses in human ischemic infarcts. Brain Pathol. 2017; doi: 10.1111/bpa.12583 [epub ahead of print] PMC6334527 29222823

[22] Schuh C , Wimmer I , Hametner S , Haider L , Van Dam AM , Liblau RS , Smith KJ , Probert L , Binder CJ , Bauer J , Bradl M , Mahad D , Lassmann H . Oxidative tissue injury in multiple sclerosis is only partly reflected in experimental disease models. Acta Neuropathol 2014; 128: 247–66 24622774 10.1007/s00401-014-1263-5 PMC4102830

[23] Cunningham C , Wilcockson DC , Campion S , Lunnon K , Perry VH . Central and systemic endotoxin challenges exacerbate the local inflammatory response and increase neuronal death during chronic neurodegeneration. J Neurosci 2005; 25: 9275–84 16207887 10.1523/JNEUROSCI.2614-05.2005 PMC6725757

[24] Holmes C , El‐Okl M , Williams AL , Cunningham C , Wilcockson D , Perry VH . Systemic infection, interleukin 1beta, and cognitive decline in Alzheimer's disease. J Neurol Neurosurg Psychiatry 2003; 74: 788–9 12754353 10.1136/jnnp.74.6.788 PMC1738504

[25] Dunn N , Mullee M , Perry VH , Holmes C . Association between dementia and infectious disease ‐ Evidence from a case‐control study. Alzheimer Dis Assoc Disord 2005; 19: 91–4 15942327 10.1097/01.wad.0000165511.52746.1f

[26] Wolf SA , Boddeke HW , Kettenmann H . Microglia in physiology and disease. Annu Rev Physiol 2017; 79: 619–43 27959620 10.1146/annurev-physiol-022516-034406

[27] Galatro TF , Holtman IR , Lerario AM , Vainchtein ID , Brouwer N , Sola PR , Veras MM , Pereira TF , Leite REP , Moller T , Wes PD , Sogayar MC , Laman JD , den Dunnen W , Pasqualucci CA , Oba‐Shinjo SM , Boddeke E , Marie SKN , Eggen BJL . Transcriptomic analysis of purified human cortical microglia reveals age‐associated changes. Nat Neurosci 2017; 20: 1162–71 28671693 10.1038/nn.4597

[28] Sandiego CM , Gallezot JD , Pittman B , Nabulsi N , Lim K , Lin SF , Matuskey D , Lee JY , O'Connor KC , Huang Y , Carson RE , Hannestad J , Cosgrove KP . Imaging robust microglial activation after lipopolysaccharide administration in humans with PET. Proc Natl Acad Sci U S A 2015; 112: 12468–73 26385967 10.1073/pnas.1511003112 PMC4603509

[29] Tronel C , Largeau B , Santiago Ribeiro MJ , Guilloteau D , Dupont AC , Arlicot N . Molecular targets for PET imaging of activated microglia: the current situation and future expectations. Int J Mol Sci 2017; 18: 802 10.3390/ijms18040802 PMC5412386 28398245

[30] Dal‐Pizzol F , Ritter C , Cassol OJ Jr , Rezin GT , Petronilho F , Zugno AI , Quevedo J , Streck EL . Oxidative mechanisms of brain dysfunction during sepsis. Neurochem Res 2010; 35: 1–12 19680806 10.1007/s11064-009-0043-4

[31] Sharshar T , Gray F , deLorin la Grandmaison G , Hopkinson NS , Ross E , Dorandeu A , Orlikowski D , Raphael JC , Gajdos P , Annane D . Apoptosis of neurons in cardiovascular autonomic centres triggered by inducible nitric oxide synthase after death from septic shock. Lancet (London, England) 2003; 362: 1799–805 10.1016/s0140-6736(03)14899-4 14654318

[32] Sharshar T , Carlier R , Bernard F , Guidoux C , Brouland JP , Nardi O , de la Grandmaison GL , Aboab J , Gray F , Menon D , Annane D . Brain lesions in septic shock: a magnetic resonance imaging study. Intensive Care Med 2007; 33: 798–806 17377766 10.1007/s00134-007-0598-y

[33] Taccone FS , Castanares‐Zapatero D , Peres‐Bota D , Vincent JL , Berre J , Melot C . Cerebral autoregulation is influenced by carbon dioxide levels in patients with septic shock. Neurocrit Care 2010; 12: 35–42 19806473 10.1007/s12028-009-9289-6

[34] Vogel DY , Vereyken EJ , Glim JE , Heijnen PD , Moeton M , van der Valk P , Amor S , Teunissen CE , van Horssen J , Dijkstra CD . Macrophages in inflammatory multiple sclerosis lesions have an intermediate activation status. J Neuroinflammation 2013; 10: 809 10.1186/1742-2094-10-35 PMC3610294 23452918

[35] Serrats J , Schiltz JC , Garcia‐Bueno B , van Rooijen N , Reyes TM , Sawchenko PE . Dual roles for perivascular macrophages in immune‐to‐brain signaling. Neuron 2010; 65: 94–106 20152116 10.1016/j.neuron.2009.11.032 PMC2873837

[36] Krasemann S , Madore C , Cialic R , Baufeld C , Calcagno N , El Fatimy R , Beckers L , O'Loughlin E , Xu Y , Fanek Z , Greco DJ , Smith ST , Tweet G , Humulock Z , Zrzavy T , Conde‐Sanroman P , Gacias M , Weng Z , Chen H , Tjon E , Mazaheri F , Hartmann K , Madi A , Ulrich JD , Glatzel M , Worthmann A , Heeren J , Budnik B , Lemere C , Ikezu T , Heppner FL , Litvak V , Holtzman DM , Lassmann H , Weiner HL , Ochando J , Haass C , Butovsky O . The TREM2‐APOE pathway drives the transcriptional phenotype of dysfunctional microglia in neurodegenerative diseases. Immunity 2017; 47: 566–581.e9 28930663 10.1016/j.immuni.2017.08.008 PMC5719893

[37] Butovsky O , Jedrychowski MP , Cialic R , Krasemann S , Murugaiyan G , Fanek Z , Greco DJ , Wu PM , Doykan CE , Kiner O , Lawson RJ , Frosch MP , Pochet N , Fatimy RE , Krichevsky AM , Gygi SP , Lassmann H , Berry J , Cudkowicz ME , Weiner HL . Targeting miR‐155 restores abnormal microglia and attenuates disease in SOD1 mice. Ann Neurol 2015; 77: 75–99 25381879 10.1002/ana.24304 PMC4432483

[38] Haynes SE , Hollopeter G , Yang G , Kurpius D , Dailey ME , Gan WB , Julius D . The P2Y12 receptor regulates microglial activation by extracellular nucleotides. Nat Neurosci 2006; 9: 1512–9 17115040 10.1038/nn1805

